# Sectional Anatomy Quiz II

**DOI:** 10.22038/aojnmb.2017.9879

**Published:** 2018

**Authors:** Rashid Hashmi

**Affiliations:** Rural Clinical School, University of New South Wales, Wagga Wagga, New South Wales, Australia

**Keywords:** Anatomy, Computed tomography, Thorax

## Abstract

This mage-based series comprises of a quiz pertaining to the identification of salient and important anatomical structures and landmarks expected to be seen at a given level on the computed tomography (CT). The representative image is followed by a series of images showing the examples of different commonly encountered pathological entities that can be seen at this level in a routine clinical practice. Readers are encouraged to identify the highlighted anatomical structures and landmarks in all the images and appreciate the alterations in the appearance of the normal structures resulting from the presence of a pathology. It is expected that this series will assist in improving the confidence of the nuclear physicians in the interpretation of the CT component of the single photon emission computed tomography (SPECT) and positron emission tomography (PET) studies.

**Figure 1:**
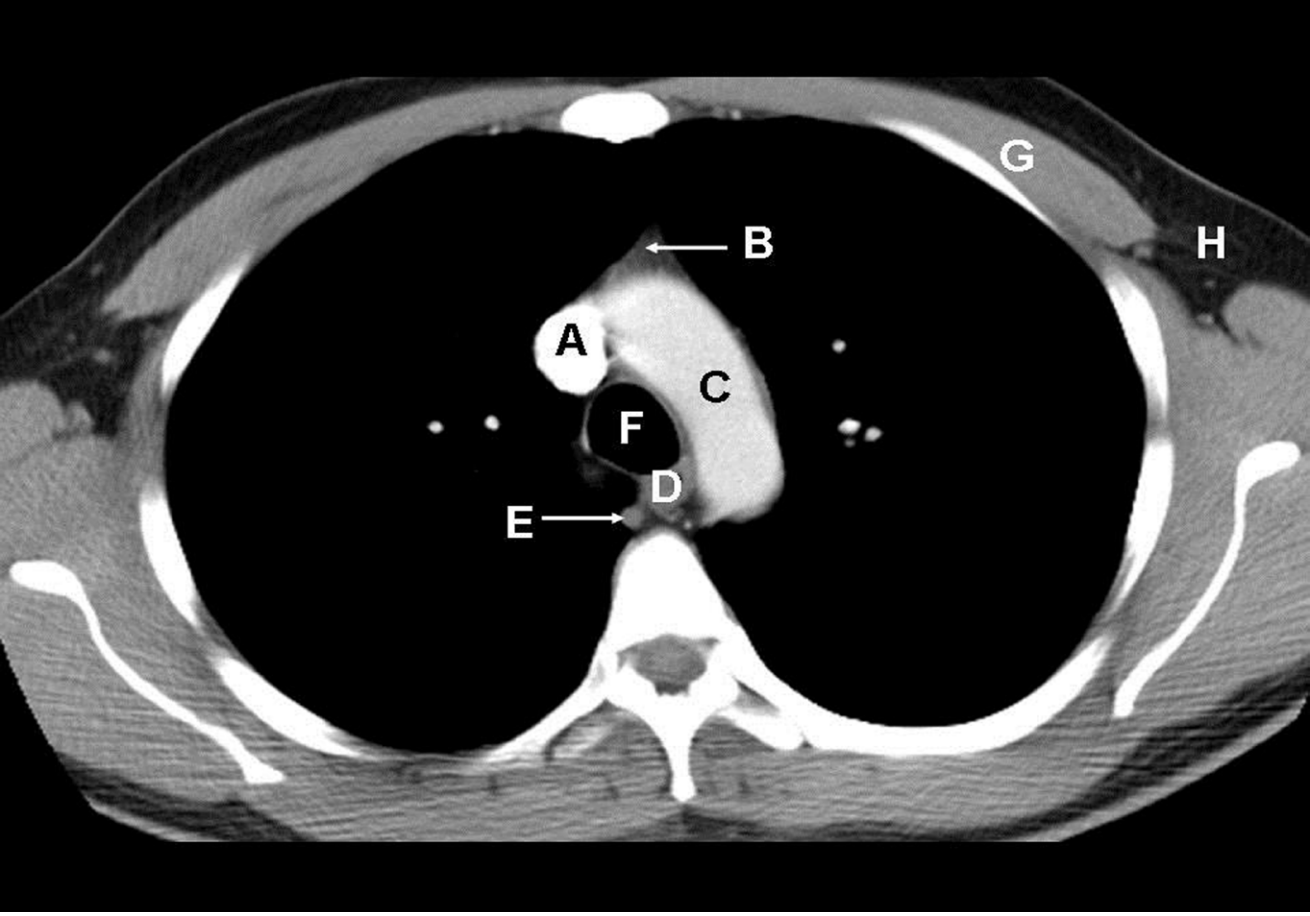
On the postcontrast-enhanced axial CT image of the chest of a 27-year-old man shown above, identify the labelled normal anatomical structures.

**Figure 2:**
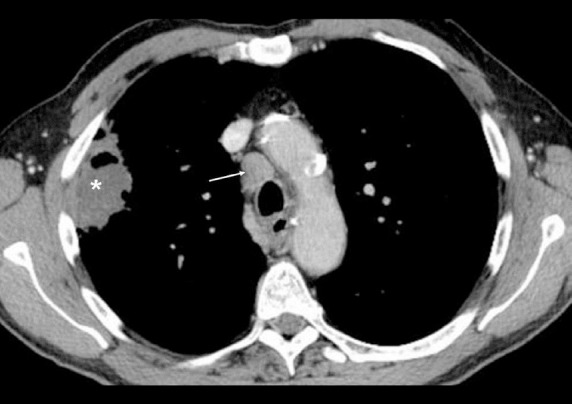
Contrast-enhanced CT scan of the chest obtained at the level of aortic arch shows a large metastatic lymph node (arrow) anterior and slightly right to the trachea (right lower paratracheal node) and a mass with irregular margins (asterisk) in the peripheral portion of the right upper lobe along the chest wall. Note the age-related calcification in the aortic arch. It is important to remember that is not uncommon to see a few normal sized (<1 cm) lymph nodes in the paratracheal region.

**Figure 3:**
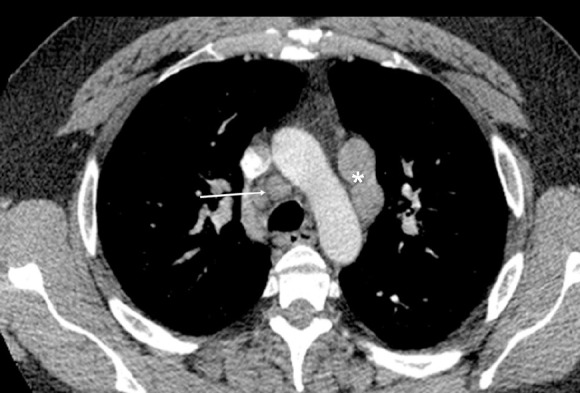
Contrast-enhanced CT of the chest obtained in a patient with Hodgkin lymphoma reveals multiple enlarged lymph nodes in the mediastinum. The lymph nodes located anterior and to the left of the aortic arch (asterisk) are called para-aortic nodes, while those located anterior and to the right of the trachea (arrow) are right lower paratracheal nodes. Note the slightly prominent but normal triangular-shaped thymus anterior to the aortic arch. The linear hypodense line seen at the junction of the anterior one-third and posterior two-third of the thoracic vertebra is partial volume effect, resulting from intervertebral disk.

**Figure 4:**
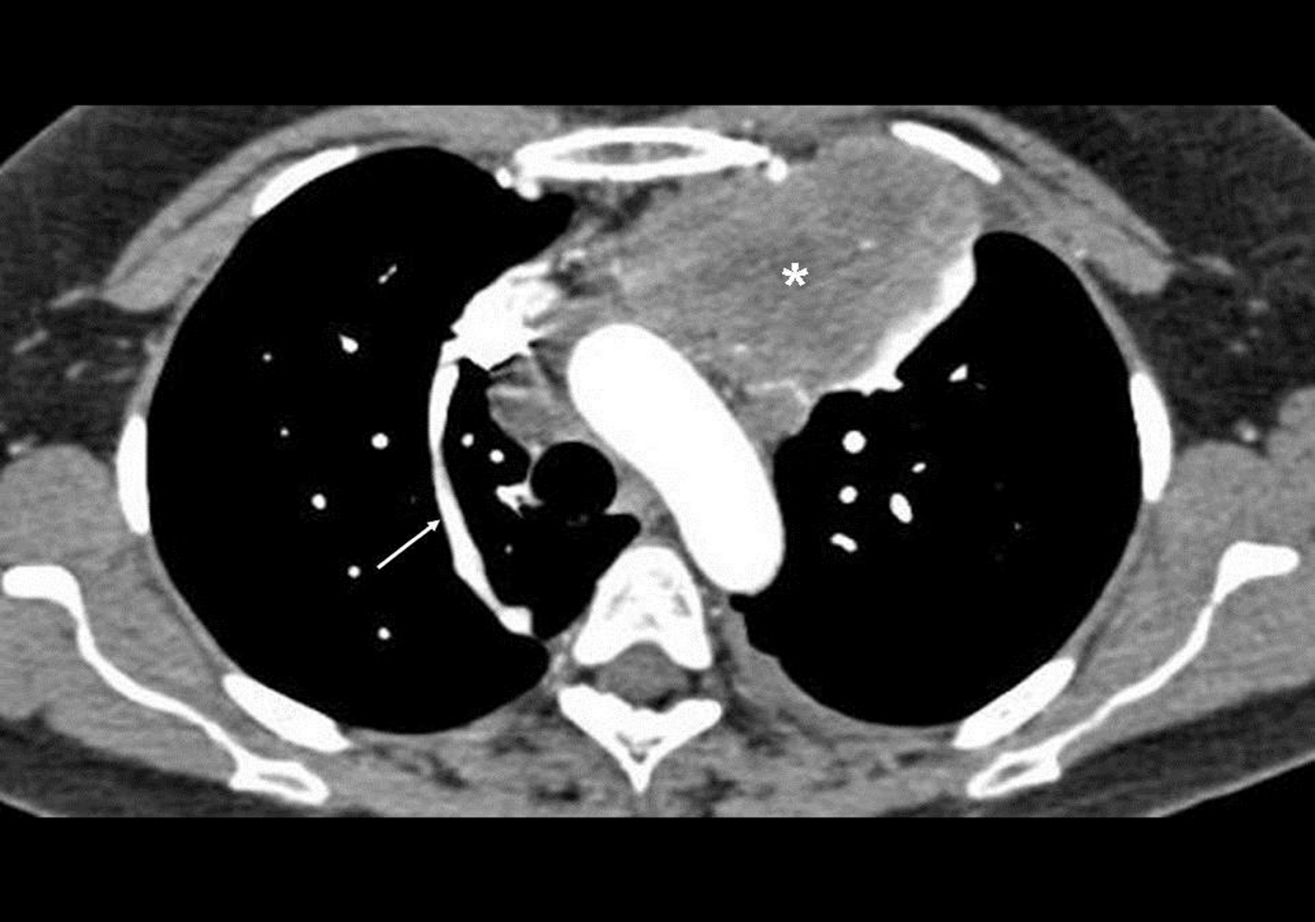
Contrast-enhanced CT of the chest of an 18-year-old shows a large anterior mediastinal mass (asterisk), which was subsequently diagnosed to be a germ cell tumour. The linear vascular structure (arrow) entering the posterior aspect of the superior vena cava is the azygous vein. It is to be noted that the appearance of azygous vein can vary and is dependent not only on its course, but also on the direction of the X-ray beam.

**Figure 5:**
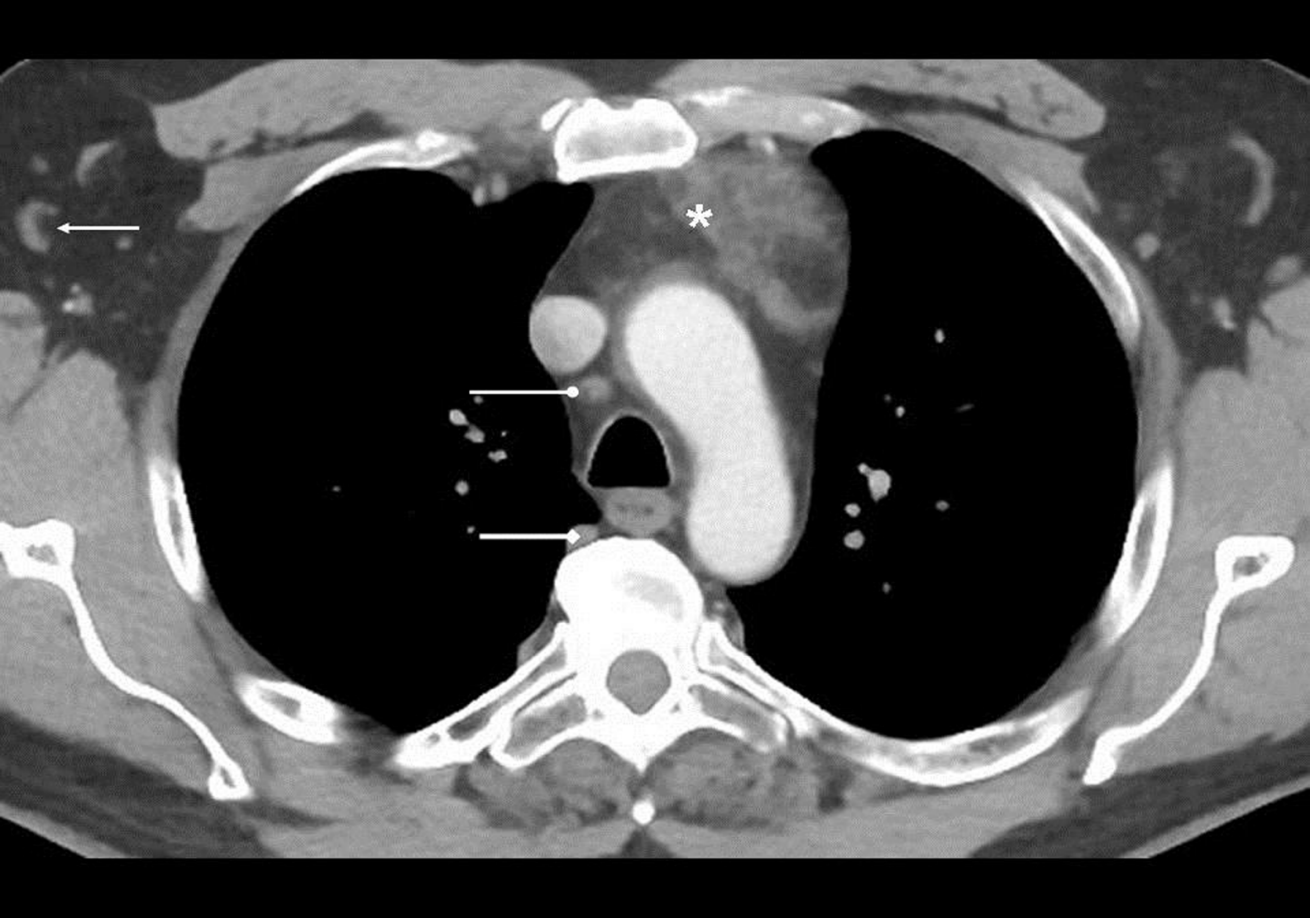
Contrast-enhanced CT of the upper chest in a 45-year-old man reveals a large heterogeneous anterior mediastinal mass (asterisk) with internal fatty density suggesting thymolipoma. A normal lymph node with a fatty hilum (arrow) is seen in the right axilla. Another normal sized lymph node (oval arrow) is seen in the right lower paratracheal region. The small circular soft tissue density seen in the right pre-vertebral region (diamond arrow) is azygous vein.

**Figure 6:**
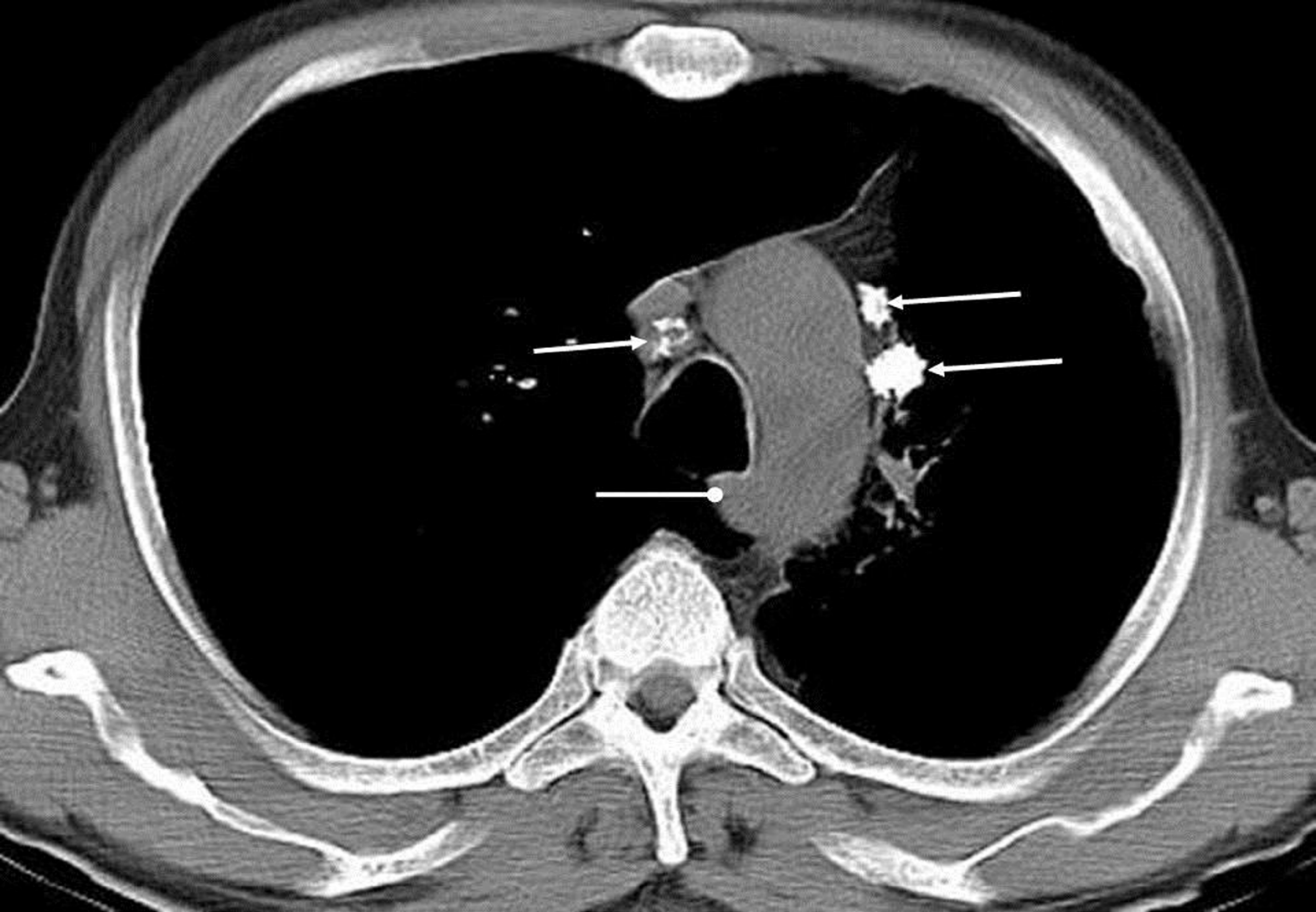
Non-contrast CT of the chest of a 65-year-old female with sarcoidosis shows multiple areas of focal calcification suggesting calcified lymph nodes (arrows) in the para-aortic and paratracheal regions of the mediastinum. The left ward deviation of the mediastinum is the result of fibrotic changes in the left lung, which can be best appreciated on the lung window. Soft tissue density seen posterior to the left side of the trachea (oval arrow) is oesophagus, which, when collapsed, can be difficult to identify.

Silicosis, tuberculosis, histoplasmosis, and treated lymphoma are few other common causes of calcified lymph nodes in the mediastinum.

**Figure 7:**
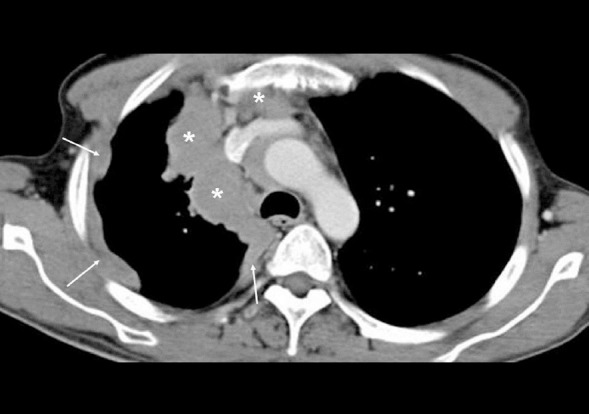
Contrast-enhanced CT of the chest of a 51-year-old male with invasive thymoma and pleural metastases is shown above. Thymoma appears as a lobulated mediastinal mass (asterisks) encircling the superior vena cava. The thickening seen around the lateral and medical aspect of the right hemithorax (arrows) represents pleural metastases.

## Points to Remember

Aortic arch is an important landmark on the axial images of the upper chest, which can be easily identified given its characteristic appearance. The anterior aspect of the arch is located anterior and to the right of the trachea, while posterior arch usually lies anterior and lateral to the thoracic spine.
Superior vena cava, at the level of the aortic arch, is seen anterior and to the right of the trachea. It is generally elliptical in shape; however, its appearance can vary due to changes in its caliber. The density of this vena cava is dependent not only on the time of the acquisition of post-contrast images, but also on the contrast injection site.
Esophagus is readily identified posterior and slightly to the left of the trachea. When collapsed, it appears as a soft tissue density structure, but has varying appearance when distended. Small amounts of air or fluid may be seen in its lumen.
Azygous vein is visualized as a small circular soft tissue density on the right side of the esophagus.
Aortic arch on the left, superior vena cava on the right, and trachea posteriorly define a somewhat triangular space called the pretracheal space. This fat-filled space contains normal sized (<1 cm) lymph nodes.
Triangular space anterior to the aortic arch and superior vena cava at this level is called pre-vascular space. This constitutes anterior mediastinum. Its normal contents include thymus, small lymph nodes, and fat. Therefore, a focal mass in this region is considered an anterior mediastinal mass. Germ cell tumours, thymic pathologies (cysts, hyperplasia, thymoma, thymic carcinoma, etc.), retrosternal extension of the thyroid gland (intrathoracic goiter), and lymphadenopathy (secondary to lymphoma, metastases, etc.) are the main pathologies encountered in this region.

### Answer

The image is at the level of the aortic arch through the superior mediastinum.

A: Superior vena cava
B: Thymus
C: Aortic arch
D: Esophagus
E: Azygous vein
F: Trachea
G: Pectoralis major muscle
H: Axilla

